# PATHWAYS LINKING PARENTAL DEATH BEFORE 18 TO ACCELERATED EPIGENETIC AGING

**DOI:** 10.1093/geroni/igad104.0719

**Published:** 2023-12-21

**Authors:** Mateo Farina, Eric Klopack, Eileen Crimmins

**Affiliations:** University of Texas at Austin, Austin, Texas, United States; University of Southern California, Los Angeles, California, United States; University of Southern California, Los Angeles, California, United States

## Abstract

Parental death before 18 is one of the most challenging adverse events that can lead to poor health in later life. Accelerated biological aging may be a key physiological mechanism that underlies this association. Data come from the Health and Retirement Study: the core surveys and the 2016 Venous Blood Study (VBS). We used second- and third- generation epigenetic clocks (GrimAge, PhenoAge, DunedinPace) to evaluate accelerated aging. Parental death before 18 and other covariates were obtained from the core surveys. To assess direct and indirect pathways, we used Structural Equation Modeling with educational attainment, depressive symptoms, and adult health behaviors (smoking, drinking, and BMI) as potential adulthood pathways that link parental death before 18 to accelerated aging. Parental death before 18 was associated with .79 years of accelerated aging. We found evidence of an indirect pathway through adulthood depressive symptoms. We did not find evidence of indirect pathways through educational attainment or adulthood wealth despite their strong negative associations with parental death before 18. Parental death before 18 was associated with accelerated epigenetic aging, which indicates that epigenetic aging may be a a key biological mechanism that links parental death in early life to health in older adulthood. Some of the association may be reduced by addressing mental health challenges that are associated with this traumatic event.

